# Compared to commercially insured patients, Medicare advantage patients adopt newer diabetes drugs more slowly and adhere to them less

**DOI:** 10.1002/edm2.245

**Published:** 2021-04-02

**Authors:** James H Flory, Jing Li, Ghadeer K. Dawwas, Charles E. Leonard

**Affiliations:** ^1^ Department of Subspecialty Medicine Endocrinology Service Memorial Sloan Kettering Cancer Center New York NY USA; ^2^ Department of Population Health Sciences Weill Cornell Medicine New York NY USA; ^3^ Department of Biostatistics, Epidemiology, and Informatics Center for Pharmacoepidemiology Research and Training Perelman School of Medicine University of Pennsylvania Philadelphia PA USA

**Keywords:** adherence, diabetes, insurance

## Abstract

**Aims:**

To compare rates of use and adherence for newer versus older second‐line diabetes drug classes in commercially insured, Medicare Advantage and dual‐eligible (covered by both Medicare and Medicaid) patients.

**Materials and Methods:**

Longitudinal cohort study using insurance claims data from 1/1/2012 to 12/31/2016 to identify patients with a first prescription, after metformin, of a second‐line diabetes drug (eg sulphonylurea, DPP‐4 inhibitor, thiazolidinedione, SGLT‐2 inhibitor or GLP‐1 receptor agonist) and to estimate their adherence to that drug class. Univariate analysis and multivariable logistic regression were used to examine the association between insurance type and use of each drug class, and between insurance type and adherence to each drug class.

**Results:**

The study population included 96,663 patients. Trends in drug use differed by insurance type. For example, sulphonylurea use declined among the commercially insured (from 46% to 39%, *p* < .001) but not among Medicare Advantage or dual‐eligible patients. Patterns of adherence also differed between insurance groups. For example, compared to commercial insurance, Medicare Advantage was associated with higher adherence to sulphonylurea (odds ratio [OR] 1.32, 95% CI 1.21–1.43)) but lower adherence to SGLT‐2 inhibitors (OR 0.43 (95% CI 0.33–0.56)).

**Conclusions:**

This study finds differences in utilization and adherence for diabetes drugs across insurance types. Older medications such as sulphonylureas appear to be more used and better adhered to among Medicare Advantage recipients, while the opposite is true for newer medication classes. These findings suggest a need to personalize selection of diabetes drugs according to insurance status, particularly when adherence needs optimization.

## Graphical abstract

Patients with Medicare Advantage coverage are less likely to adhere to expensive new diabetes drugs than patients with commercial insurance. Older, less expensive diabetes drugs may continue to play an important role in the care of these patients.

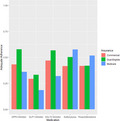

## 1 INTRODUCTION

New drugs for treatment of type 2 diabetes mellitus (T2DM) have proliferated over the past 15 years, with the introduction of glucagon‐like peptide 1 (GLP‐1) receptor agonists, dipeptidyl peptidase‐4 (DPP‐4) inhibitors and sodium‐glucose transport protein 2 (SGLT‐2 inhibitors).^1^, ^2^, ^3^, ^4^ These agents were originally promoted for having fewer risks of hypoglycaemia and weight gain than older diabetes drugs. The newer diabetes drug classes have also benefited from large recent clinical trials showing that SGLT‐2 inhibitors and GLP‐1 receptor agonists prevent major adverse cardiovascular events and progression of kidney disease.^1^, ^2^ Professional guidelines increasingly encourage use of the newer medications as preferred second‐line therapy (after first‐line use of metformin) over older agents (ie sulphonylureas, insulin and thiazolidinediones).^1^, ^2^

While adoption of newer diabetes drugs has been significant, sulphonylureas remain the most commonly used second‐line agent overall.^5^ Concerns about contraindications, safety, tolerability and cost all have the potential to hasten or slow the adoption of newer diabetes drugs in specific populations.^3^ For example, since the newer diabetes drugs have no generic equivalents and remain relatively expensive, professional guidelines note that older drug classes such as sulphonylureas and thiazolidinediones may be preferred in those patients with limited financial means, since substantial empirical evidence supports that high cost may translate into poor adherence.^1^, ^6^, ^7^, ^8^, ^9^, ^10^ At the same time, the increased risk of hypoglycaemia in older adults has led some providers to recommend adopting newer drugs more quickly in that population.^11^, ^12^

These competing considerations make it difficult to predict whether, and how quickly, newer diabetes drugs will displace older drugs (especially sulphonylureas) in any given population. For example, commercially insured patients might be expected to have generally higher incomes and lower cost sharing than Medicare patients, which could drive more rapid adoption of newer medications.^13^ On the other hand, the high cardiovascular comorbidity burden and hypoglycaemia risk seen in older patients might drive more rapid adoption of newer drugs in Medicare patients.

This study aimed to compare rates of adoption of and adherence to newer diabetes drugs in commercially insured, Medicare Advantage and dual‐eligible (covered by both Medicare and Medicaid) patients, test the hypothesis that adoption of and adherence to newer drugs is lower in Medicare patients compared to commercially insured patients and assess whether differences in utilization are explained by differences in out of pocket (OOP) drug cost.

## 2 MATERIALS AND METHODS

### 2.1 Population

This longitudinal study used the Health Care Cost Institute (HCCI) database, which merges de‐identified claims data from commercial insurance carriers in the United States.^14^ Details of individual insurance plans (eg formularies) are unavailable, but plans are identified as commercial insurance versus Medicare Advantage plans. A minority of the Medicare Advantage patients are also identified as dual‐eligible recipients of Medicaid. The data set includes medical procedure and diagnosis codes from inpatient and outpatient settings, outpatient prescription claims and basic demographic information (excluding race). Laboratory and vital sign data are unavailable.

The analysis included data from 1/1/2012 to 12/31/2016. The defining event for study entry (‘index date’) was the first prescription, after metformin, of a second‐line diabetes drug (eg sulphonylurea [glipizide, glyburide or glimepiride], DPP‐4 inhibitor [sitagliptin, saxagliptin, linagliptin or alogliptin], thiazolidinedione [rosiglitazone or pioglitazone], GLP‐1 receptor agonist [exenatide, dulaglutide, lixisenatide or liraglutide] or SGLT‐2 inhibitor [canagliflozin, empagliflozin or dapagliflozin]). Prescriptions for a second‐line (non‐metformin) drug could also include insulin, meglitinides [nateglinide or repaglinide] or an alpha‐glucosidase inhibitor [acarbose], although these drugs were excluded from most analyses due to the challenges in calculating adherence to insulin and small cohort sizes for meglitinides and alpha‐glucosidase inhibitors.

The study cohort was restricted to adult patients with: at least one metformin prescription <90 days prior to the index date; ≥1 year of baseline data prior to the index date with no use of diabetes drugs other than metformin; a non‐missing insurance status; a baseline diagnosis of diabetes mellitus based on the presence of at least one ICD‐9 or ICD‐10 code for diabetes and an identifiable 5‐digit zip code of residence. Patients were only included if initiating a single non‐metformin (ie second‐line) diabetes drug; therefore, patients beginning multiple new diabetes drugs on the same day were excluded. Patients were also excluded if the initial prescription for the second‐line drug had >30‐day supply or if a mail‐order pharmacy was used, on the assumption that such prescriptions were less likely to be true incident use. For analyses taking adherence as the outcome, patients were also required to have ≥1 year of post‐index follow‐up data.

### 2.2 Economic and demographic covariates

Covariates were assessed for each patient using data from the year prior to the index date and included: age, sex, insurance type (commercial, Medicare Advantage and Medicare/Medicaid dual‐eligible), common medical comorbidities (myocardial infarction [MI], heart failure [HF], peripheral vascular disease [PVD], stroke, dementia, complications of diabetes, liver disease, renal disease and cancer) and number of drug classes used. Five‐digit zip codes were used to link patients to the median income and racial/ethnic composition in their zip code level based on 2010 census data.^15^

### 2.3 Prescription‐related covariates

The study used two cost measures that were associated with each prescription claim: the calculated sum of payments by both patient and insurer (total cost); and the sum of out of pocket (OOP) costs. Costs reported and analysed are for the initial (index) prescription of the second‐line drugs.

### 2.4 Outcomes

Adherence was defined as the proportion of days covered (PDC) with drug supply during the first year of follow‐up, and ‘adequate secondary adherence’ (defined as fewer than 20% of days without drug supply during the first year of follow‐up).^16^ These metrics were calculated using the AdhereR package in R.^17^

### 2.5 Analysis

Baseline population characteristics and unadjusted estimates of the outcome rates across all variables were summarized using means, medians, and proportions, and chi‐square or t tests as appropriate to calculate statistical significance. Rates of use of different drug classes over time were described. Logistic regression with multivariable adjustment was used to examine the effect of insurance type and other variables on the odds of a patient receiving sulphonylurea as opposed to a newer antidiabetes medication (GLP‐1 receptor agonist, SGLT‐2 inhibitor or DPP‐4 inhibitor) after restricting the cohort to patients receiving those exposures. Logistic regression with multivariable adjustment was also used to generate odds ratios for the effect of insurance type and other variables on adherence to each medication class. In the primary analysis, OOP cost was excluded from this model as a potential mediator. In secondary analysis, it was included to assess for potential mediation.

In sensitivity analyses, 1:1 propensity score matching was used in place of logistic regression for all multivariable analyses. In additional sensitivity analysis, inclusion criteria were relaxed to permit initial prescriptions >30 days and the use of mail‐order pharmacy in the initial prescription, initial prescriptions not preceded by a diabetes diagnosis or individuals without zip code level data. Finally, for adherence analyses, the required duration of follow‐up was shortened from 12 months to 6 months.

This research was ruled exempt by the Institutional Review Board of Weill Cornell Medical College. Data are not available for distribution under the Health Care Cost Institute's terms of access to them.

## 3 RESULTS

After application of inclusion/exclusion criteria, the study population included 96,663 patients, of whom 70,503 had commercial insurance, 22,517 had Medicare Advantage, and 3643 were dual‐eligible, having both Medicare and Medicaid coverage (Supplementary Table 1). In analyses of adherence, requiring one year of follow‐up data and excluding insulin users, cohort size was further reduced to 76,359 individuals. The cohort was evenly distributed by sex except that the majority (64%) of dual‐eligible beneficiaries were female. Age distribution differed by insurance type, with Medicare Advantage patients being older. Medicare Advantage and dual‐eligible patients also had higher rates of comorbidities, and greater prescription drug use at baseline. Second‐line agent use varied by insurance type, with GLP‐1 receptor agonists and SGLT‐2 inhibitors used most frequently by commercially insured patients and sulphonylureas used most frequently by Medicare Advantage patients (Table 1).

**TABLE 1:** Baseline characteristics. SMD = standardized mean difference relative to commercially insured group

	Commercial	Dual‐eligible	SMD	Medicare	SMD
*N*	70,503	3643		22,517	
Female Sex	31,942 (45)	2333 (64)	0.38	11,329 (50)	0.10
Age					
<35	3137 (4)	39 (1)	1.33	21 (0)	2.15
35–44	11,076 (16)	133 (4)		201 (1)	
45–54	24,002 (34)	483 (13)		1022 (5)	
55–64	26,313 (37)	815 (22)		3373 (15)	
65–74	4752 (7)	1356 (37)		11,603 (52)	
75–85	990 (1)	679 (19)		5340 (24)	
85+	233 (0)	138 (4)		957 (4)	
Year					
2013	16,332 (23)	703 (19)	0.12	4655 (21)	0.08
2014	16,812 (24)	819 (22)		5051 (22)	
2015	18,659 (26)	1020 (28)		6194 (28)	
2016	18,700 (27)	1101 (30)		6617 (29)	
Baseline comorbidities					
MI	1190 (2)	185 (5)	0.19	1107 (5)	0.18
CHF	2208 (3)	606 (17)	0.46	3080 (14)	0.39
PVD	2666 (4)	562 (15)	0.40	2928 (13)	0.34
Stroke	2814 (4)	526 (14)	0.37	3172 (14)	0.36
Dementia	113 (0)	94 (3)	0.21	491 (2)	0.19
DMcx	9611 (14)	1165 (32)	0.45	6424 (29)	0.37
Liver	4923 (7)	339 (9)	0.09	1517 (7)	0.01
Renal	2370 (3)	504 (14)	0.38	3156 (14)	0.39
Cancer	3032 (4)	285 (8)	0.15	2227 (10)	0.22
Baseline drug classes					
1–3	33,794 (48)	535 (15)	0.95	5161 (23)	0.64
4	12,101 (17)	429 (12)		3389 (15)	
5–6	16,372 (23)	1144 (31)		7038 (31)	
>6	8236 (12)	1535 (42)		6929 (31)	
Median zip code income ($)					
<42,000	15,992 (23)	1807 (50)	0.78	8527 (38)	0.47
42,000–53,999	17,601 (25)	1098 (30)		6758 (30)	
43,000–70,999	18,365 (26)	516 (14)		4623 (21)	
>70,999	18,543 (26)	222 (6)		2609 (12)	
Zip code >50% White	60,817 (86)	3043 (84)	0.08	20,196 (90)	0.11
Zip code >50% Black	4914 (7)	441 (12)	0.18	1533 (7)	0.01
Diabetes drug prescribed after metformin					
Sulphonylurea (%)	28,924 (41)	1804 (50)	0.17	11,464 (51)	0.20
DPP−4 inhibitor (%)	19,825 (28)	1030 (28)	0.00	6431 (29)	0.01
TZD (%)	2545 (4)	131 (4)	0.00	949 (4)	0.03
SGLT−2 inhibitor (%)	9508 (13)	164 (5)	0.32	960 (4)	0.33
GLP−1 receptor agonist (%)	4711 (7)	139 (4)	0.13	654 (3)	0.18
Meglitinide (%)	325 (0)	22 (1)	0.02	213 (1)	0.06
Alpha‐glucosidase inhibitor (%)	105 (0)	Count <11	Count<11	46 (0)	0.01
Insulin (%)	4560 (6)	352 (10)	0.12	1800 (8)	0.06

**FIGURE 1:**
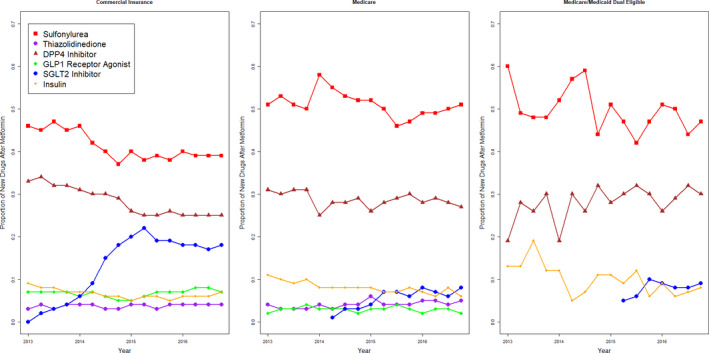
Trends in initial choice of second‐line diabetes by year by insurance type. Missing points denote 0 or very low rates of usage such that showing proportions would create cells with fewer than 11 individuals

Over time, rates of sulphonylurea use declined among the commercially insured (from 46% to 39%, *p* < .001) but not among Medicare Advantage or dual‐eligible patients. DPP‐4 inhibitor use also declined only among commercially insured patients (from 33% to 25%, *p* < .001). SGLT‐2 inhibitor use increased in all groups, from 0% in 2012 to a maximum of 9% and 8% among Medicare Advantage and dual‐eligible patients, and to a maximum of 18% among commercially insured patients. There were no large trends in use of insulin, thiazolidinediones or GLP‐1 receptor agonists over time (Figure 1).

Multivariable modelling, adjusting for calendar year, demographics, major comorbidities and zip code level variables showed persistent associations between insurance type and use of sulphonylureas as opposed to the newer agents (GLP‐1 receptor agonist, SGLT‐2 inhibitor and DPP‐4 inhibitor), with odds ratios (ORs) of 1.41 (95% CI 1.35–1.48) for Medicare Advantage and 1.41 (95% CI 1.31–1.53) for dual‐eligible patients (Table 2).

**TABLE 2:** Odds ratios for choice of sulphonylurea over DPP‐4 inhibitor, GLP‐1 receptor agonist, or SGLT‐2 inhibitor

Variable	Odds ratio	95% CI lower	95% CI upper
Commercial Insurance	Ref		
Dual‐eligible	1.41	1.31	1.53
Medicare Advantage	1.41	1.35	1.48
Female Sex	0.85	0.83	0.87
Age			
18–34	0.9	0.83	0.99
35–44	0.81	0.77	0.86
45–54	0.81	0.77	0.86
55–64	0.90	0.86	0.95
65–74	Ref		
75–84	1.17	1.1	1.25
85+	1.31	1.15	1.49
Year			
2013	Ref		
2014	0.87	0.83	0.91
2015	0.75	0.72	0.78
2016	0.78	0.75	0.81
Zip code median income			
<=42,000	Ref		
42,000 to 54,000	0.99	0.96	1.03
54,000 to 72,000	0.91	0.88	0.95
72,000+	0.77	0.74	0.80
Comorbidities			
MI	1.27	1.16	1.4
CHF	1.11	1.04	1.19
PVD	0.95	0.90	1.01
Stroke	1.05	0.99	1.12
Dementia	1.01	0.84	1.21
DMcx	1.00	0.97	1.04
Liver	1.02	0.97	1.08
Renal	1.1	1.04	1.17
Cancer	1.01	0.95	1.07
Zip Code >50% White	0.9	0.85	0.95
Zip Code >50% Black	1.02	0.95	1.10
Baseline drug classes used			
1–3	Ref		
4	0.91	0.88	0.95
5–6	0.89	0.86	0.93
>6	0.88	0.84	0.92

Total and OOP medication costs were highest for newer medications (DPP‐4 inhibitor, GLP‐1 receptor agonist, SGLT‐2 inhibitor) and insulin. Costs were lowest for older medications (sulphonylureas and thiazolidinediones). While total costs were similar across all insurance types, OOP cost was highest for Medicare Advantage and commercially insured patients and very low for dual‐eligible patients (Supplementary Table 2). Older age was independently associated with greater sulphonylurea use, while residence in a higher‐income zip code was independently associated with less sulphonylurea use.

**FIGURE 2:**
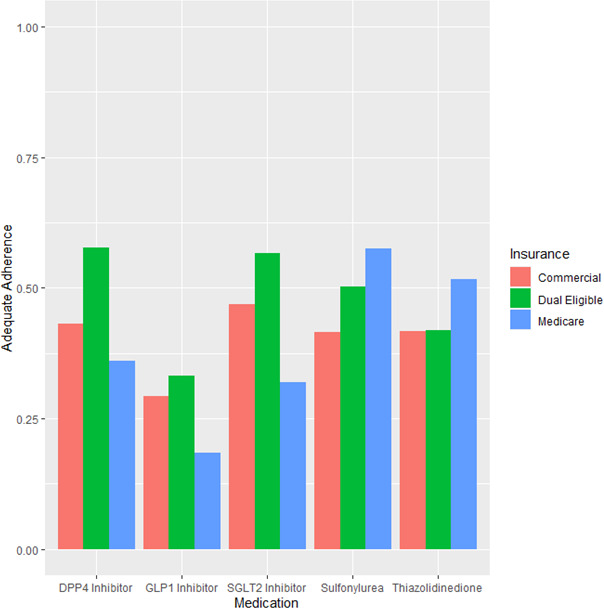
Proportion of patients with adequate adherence (proportion of days covered >0.8) by drug class and insurance type

Adherence patterns varied by insurance type, with adherence to newer agents higher among commercially insured and dual‐eligible patients and lower among Medicare Advantage patients (Figure 2). For sulphonylureas and thiazolidinediones, this pattern was reversed, with higher adherence among Medicare Advantage patients. These differences persisted after multivariable adjustment. Taking commercially insured patients as the reference group, the adjusted OR for adherence to sulphonylurea was 1.20 (95% confidence interval [CI] 1.05–1.37) for dual‐eligible patients and 1.32 (95% CI 1.21–1.43) for Medicare patients. For DPP‐4 inhibitor, it was 1.49 (95% CI 1.25–1.79) for dual‐eligible patients and 0.59 (0.53–0.66) for Medicare patients. For GLP‐1 receptor agonist, it was 1.17 (95% CI 0.72–1.90) for dual‐eligible patients and 0.41 (95% CI 0.29–0.59) for Medicare patients. For SGLT‐2 inhibitor, it was 1.29 (95% CI 0.75–2.19) for dual‐eligible patients and 0.43 (95% CI 0.33–0.56) for Medicare patients. For thiazolidinediones, it was 1.18 (95% CI 0.69–2.04) for dual‐eligible patients and 1.14 (95% CI 0.85–1.55) for Medicare patients. Inclusion of OOP cost in the model to assess for mediation had little effect, except that the association between dual‐eligible patients and higher adherence to DPP‐4 inhibitors was eliminated (Supplementary Table 3).

Sensitivity analyses using propensity score matching in place of regression, or relaxing inclusion criteria to include patients without baseline diabetes diagnosis or with an initial prescription for longer than 30 days or for mail‐order pharmacy did not materially alter these results (data not shown). Shortening the required follow‐up period from 12 to 6 months for adherence analyses did not alter those results materially either (data not shown). Propensity score matching failed to achieve balance on many covariates, with standardized mean differences >0.2 (Supplementary Table 4).

## 4 DISCUSSION

This study finds substantial differences in patterns of utilization and adherence for diabetes drugs across patients with different types of insurance. These populations are very different from one another, and the data are insufficient to prove or disprove the hypothesis that drug cost mediates these differences. Nonetheless, this finding has practical implications for research and practice, and opens potential new avenues for research into the comparative safety and effectiveness of newer diabetes drug classes.

From a prescriber perspective, these results suggest that Medicare Advantage patients on average adhere best to older drug classes. Figure 2 illustrates this most clearly, showing that Medicare Advantage patients are most adherent to sulphonylureas and thiazolidinediones, generic drug classes in use since the 20th century, and least adherent to the newer SGLT‐2 inhibitor, GLP‐1 receptor agonist and DPP‐4 inhibitor classes. For commercially insured and dual‐eligible patients, this pattern is reversed. Consistent with this, Figure 1 shows steady rates of use of sulphonylureas among Medicare Advantage patients, in whom they remain the most commonly used second‐line drug class, and no rapid increases in use of newer drug class. In contrast, for the commercially insured, sulphonylurea use is declining while SGLT‐2 inhibitor use rises.

Even with limited insight into the causes of these patterns, they have immediate utility to providers. Despite guidelines suggesting that sulphonylureas are an outdated treatment for type 2 diabetes, providers may still need to consider sulphonylureas or thiazolidinediones in older, Medicare‐insured patients for whom adherence is a concern. Conversely, if providers use SGLT‐2 inhibitors in this population, careful follow‐up and attention to adherence is warranted.

From a research and policy perspective, these findings may help to explain why rates of hypoglycaemia have not trended down over time in Medicare patients despite the availability of newer drugs that do not cause hypoglycaemia.^18^ It also suggests opportunities to exploit the different rates of adoption of new drugs across different populations as a natural experiment. For example, the risk‐benefit profile of SGLT‐2 inhibitors may potentially be better understood by monitoring rates of changes in outcomes like ketoacidosis and HF admissions among commercially insured patients (who have rapidly rising exposure to SGLT‐2 inhibitors) compared to Medicare patients (who are adopting this drug class much more slowly).^19^

The causes of these differences in adherence and drug utilization across insurance types need further study. A secondary hypothesis of this study was that higher levels of cost sharing for expensive newer drugs might mediate lower use by Medicare Advantage patients compared to other types of insurance. These analyses did not support that hypothesis, both because OOP cost of drugs were similar between Medicare Advantage and commercially insured patients, and because inclusion of OOP cost in multivariable analysis as a potential mediator did not eliminate observed differences in adherence.

However, cost may still play a key role in the differences observed. One major limitation of this data set is that it cannot show the cost of prescriptions that are never filled, which may lead to substantial underestimates of how often high drug costs prevent drug use entirely, so‐called ‘primary nonadherence’.^15^ A related issue is that cost may vary across a calendar year, particularly for Medicare patients who encounter coverage gaps, so that the observed cost of an initial prescription may not be reflective of cost for subsequent (potentially unfilled) prescriptions. Indeed, an analysis limited to Medicare Part D patients leveraged this phenomenon to conduct a difference‐in‐difference analysis and did find that adherence to more expensive diabetes drugs appeared to drop when patients entered a coverage gap and the OOP cost of the drugs increased.^10^

Many other possible mechanisms also need to be considered. The three insured groups are profoundly different on measured covariates with known effects on adherence, such as age and major comorbidities. These populations likely differ significantly on unmeasured covariates such as individual income, although a more limited cross‐sectional analysis found that patients with Medicare were less likely to use expensive diabetes drugs independent of their income.^20^ Details of benefit design such as formulary restrictions and prior authorization policies are not available, nor are other potentially relevant measures of access, such as whether a patient's provider is an endocrinologist. Due to these limitations, this is a descriptive study, rather than an effort at causal inference.

This research has other limitations. First, this claims database does not capture important patient variables, including race, body mass index and HbA1c. Second, although the requirement of a baseline diagnosis code for type 2 diabetes as well as metformin use likely excluded most cases of type 1 and gestational diabetes, residual rates of those other phenotypes might be another difference between insurance groups. Third, factors that might have provided further insight into the phenomena observed, such as adherence to baseline medications and rates of progression to a third diabetes drug, were not examined. Fourth, analyses of adherence, which required one year of follow‐up data, are susceptible to immortal time bias, although shortening the required follow‐up period to 6 months to minimize such bias did not affect study results. Finally, use of claims data to measure adherence has additional limitations—for example, acute events such as hospitalization may result in periods when patients are appropriately not using their home medication supply.

Another consideration important to interpreting these results is that the study period covers an era of rapid evolution in evidence and practice in diabetes treatment. In 2012, SGLT‐2 inhibitors were not available and the guidelines did not cite cardiovascular benefits from DPP‐4 inhibitors or GLP‐1 receptor agonists, while raising concerns about their long‐term safety.^21^ The intervening period included publication of landmark cardiovascular outcome studies and multiple approvals of new SGLT‐2 inhibitors and GLP‐1 receptor agonists. By 2016, ADA guidelines cited cardiovascular benefit from SGLT‐2 inhibitors and reduction in cardiovascular risk factors from GLP‐1 receptor agonists as advantages, and by 2021 explicitly favoured these drug classes for large subgroups of patients with diabetes.^22^ As it now appears that limitations on access and adherence to the newer diabetes drugs may result in inferior care, these findings are both highly relevant and in need of replication in data more recent than the end of 2016.

In summary, these descriptive analyses provide actionable information. Adoption of SGLT‐2 inhibitors has been far more rapid among commercially insured patients than among Medicare Advantage or dual‐eligible patients, and sulphonylureas remain particularly widely used among older and Medicare Advantage patients. These findings have direct relevance to research and clinical practice.

## CONFLICT OF INTEREST

CEL serves on the Executive Committee of the University of Pennsylvania’s Center for Pharmacoepidemiology Research and Training. The Center receives unrestricted support for education from Pfizer. JHF has consulted for Boehringer Ingelheim and Eli Lilly and Company. No authors have other conflicts of interest to disclose.

## Supporting information

Supplementary Material

## Data Availability

Data are not available for distribution under the Health Care Cost Institute's terms of access to them.
