# Supplementary material for: Compared to commercially insured patients, Medicare advantage patients adopt newer diabetes drugs more slowly and adhere to them less
Source: Endocrinol Diabetes Metab. 2021 Apr 2;4(3):e00245. doi: 10.1002/edm2.245 (PMC8279610; doi:10.1002/edm2.245)
Supplement: Supplementary file 1 — Supplementary Material [file EDM2-4-e00245-s001.docx]

**Supplementary Data**

|  |  |
| --- | --- |
| **All second line drug starts** | **1,860,276** |
| **>90 days of metformin use prior to second line start** | **313,948** |
| **Baseline year of data available** | **186,016** |
| **Zip-code level data available** | **179,686** |
| **Baseline diabetes diagnosis** | **138,764** |
| **30 day initial supply of medication** | **96,663** |
| **Year of follow-up data available** | **76,359** |

Supplementary Table 1. Attrition.

|  | **Commercial Insurance** | | **Medicare** | | **Dual-eligible** | |
| --- | --- | --- | --- | --- | --- | --- |
| **Drug** | **Out of Pocket ($)** | **Total ($)** | **Out of Pocket ($)** | **Total ($)** | **Out of Pocket ($)** | **Total ($)** |
| **DPP-4 Inhibitor** | 60.16 | 394.36 | 71.25 | 495.67 | 5.19 | 494.25 |
| **GLP-1 Receptor Agonist** | 72.58 | 565.03 | 81.78 | 728.01 | 5.57 | 740.08 |
| **Insulin** | 64.04 | 339.11 | 66.47 | 353.41 | 3.87 | 306.11 |
| **SGLT-2 Inhibitor** | 85.47 | 410.65 | 84.08 | 531.29 | 5.61 | 574.14 |
| **Sulfonylurea** | 7.45 | 9.51 | 4.66 | 10.78 | 1.36 | 9.87 |
| **Thiazolidinedione** | 18.72 | 57.60 | 12.49 | 34.38 | 1.93 | 35.51 |

Supplementary Table 2. Drug costs (out of pocket and total) for index prescriptions by insurance type

|  | Sulfonylurea | DPP-4 Inhibitor | GLP-1 Receptor Agonist | SGLT-2 Inhibitor | Thiazolidinedione |
| --- | --- | --- | --- | --- | --- |
| **Unadjusted** |  |  |  |  |  |
| Commercial | Ref | ref | ref | Ref | Ref |
| Dual-eligible | 1.45 (1.28-1.64) | 1.70 (1.44-2.00) | 1.20 (0.77-1.88) | 1.18 (0.71-1.97) | 1.11 (0.69-1.79) |
| Medicare | 1.81 (1.71-1.92) | 0.79 (0.73-0.85) | 0.50 (0.38-0.66) | 0.49 (0.39-0.60) | 1.45 (1.18-1.77) |
| **Adjusted (OOP and total cost excluded)** |  |  |  |  |  |
| Commercial | Ref | ref | ref | Ref | Ref |
| Dual-eligible | 1.20 (1.05-1.37) | 1.49 (1.25-1.79) | 1.17 (0.72-1.90) | 1.29 (0.75-2.19) | 1.18 (0.69-2.04) |
| Medicare | 1.32 (1.21-1.43) | 0.59 (0.53-0.66) | 0.41 (0.29-0.59) | 0.43 (0.33-0.56) | 1.14 (0.85-1.55) |
| **Adjusted OOP cost included, total cost excluded)** |  |  |  |  |  |
| Commercial | Ref | ref | ref | Ref | ref |
| Dual-eligible | 1.19 (1.04-1.37) | 1.08 (0.87-1.33) | 0.87 (0.49-1.54) | 1.34 (0.74-2.42) | 1.21 (0.67-2.18) |
| Medicare | 1.30 (1.20-1.41) | 0.67 (0.60-0.75) | 0.42 (0.29-0.60) | 0.44 (0.34-0.58) | 1.16 (0.85-1.58) |

Supplementary Table 3. Odds ratios for adherence defined by proportion of days covered greater than 80%. Adjusted analyses adjust for all covariates listed in table 3 as well as for cost variables as described in the row header.

|  | Commercial | Medicare | SMD |
| --- | --- | --- | --- |
| N | 9301 | 9301 |  |
| Zip Code Income |  |  | 0.093 |
| <= 42,000 | 2860 (31) | 3164 (34) |  |
| 42,000 to 54,000 | 2372 (26) | 2442 (26) |  |
| 54,000 to 72,000 | 2139 (23) | 2044 (22) |  |
| 72,000+ | 1930 (21) | 1651 (18) |  |
| Age |  |  | 0.302 |
| <35 | 51 ( 1) | 18 ( 0) |  |
| 35-44 | 250 ( 3) | 164 ( 2) |  |
| 45-54 | 885 (10) | 888 (10) |  |
| 55-64 | 2910 (31) | 2836 (30) |  |
| 65-74 | 4144 (45) | 3372 (36) |  |
| 75-85 | 856 ( 9) | 1665 (18) |  |
| 85+ | 205 ( 2) | 358 ( 4) |  |
| Female Sex | 4424 (48) | 4382 (47) | 0.009 |
| Year |  |  | 0.034 |
| 2013 | 2132 (23) | 2043 (22) |  |
| 2014 | 2095 (23) | 2116 (23) |  |
| 2015 | 2518 (27) | 2467 (27) |  |
| 2016 | 2556 (27) | 2675 (29) |  |
| MI | 300 ( 3) | 484 ( 5) | 0.099 |
| CHF | 717 ( 8) | 1301 (14) | 0.203 |
| PVD | 827 ( 9) | 1295 (14) | 0.159 |
| Stroke | 864 ( 9) | 1313 (14) | 0.151 |
| Dementia | 63 ( 1) | 174 ( 2) | 0.107 |
| DMcx | 2076 (22) | 2637 (28) | 0.139 |
| Liver | 645 ( 7) | 778 ( 8) | 0.054 |
| Renal | 809 ( 9) | 1314 (14) | 0.171 |
| Cancer | 759 ( 8) | 998 (11) | 0.088 |
| **Zip Code > 50% White** | 8268 (89) | 8121 (87) | 0.049 |
| **Zip Code > 50% Black** | 617 ( 7) | 717 ( 8) | 0.042 |
| **Baseline Drug Classes Used** |  |  | 0.159 |
| 1-3 | 2871 (31) | 2303 (25) |  |
| 4 | 1465 (16) | 1464 (16) |  |
| 5-6 | 2718 (29) | 2763 (30) |  |
| >6 | 2247 (24) | 2771 (30) |  |

Supplementary Table 4. Baseline characteristics after nearest neighbor propensity score matching, caliper width 0.2 SMD= standardized mean difference.
